# Delayed Gastric Emptying and Other Adverse Outcomes in Patients Undergoing Classic Whipple Versus Pylorus-Sparing Pancreatoduodenectomy

**DOI:** 10.7759/cureus.69406

**Published:** 2024-09-14

**Authors:** Cynthia Martinez-Cabrera, Alejandro Martinez-Esteban, Natalia M Barron-Cervantes, Alfonso Bandin-Musa, Carlos Chan

**Affiliations:** 1 General and Gastrointestinal Surgery Service, Fundacion Clinica Medica Sur, Mexico City, MEX; 2 Member of the Mexican Faculty of Medicine, Universidad La Salle Mexico, Mexico City, MEX; 3 General and Gastrointestinal Surgery Service, Fundación Clínica Medica Sur, Mexico City, MEX; 4 Hepato-Pancreato-Biliary Surgery, Fundacion Clinica Medica Sur, Mexico City, MEX; 5 Hepato-Pancreato-Biliary Surgery, Instituto Nacional de Ciencias Medicas y Nutricion Salvador Zubiran, Mexico City, MEX

**Keywords:** complications, delayed gastric emptying, pancreatoduodenectomy, post surgical outcomes, pylorus-sparing transverso-longmire technique, whipple

## Abstract

Background and objective

Pancreatoduodenectomy (PD), or the Whipple procedure, has many variants. There is a lack of data in the literature in terms of comparing various types of this procedure. This study aimed to compare the classic Whipple procedure with the pylorus-sparing Transverso-Longmire technique, focusing on postoperative complications and delayed gastric emptying (DGE).

Methodology

We conducted a retrospective observational study including 97 adult patients who underwent either the classic Whipple procedure or the pylorus-sparing technique at Hospital Médica Sur from 2016 to 2021. Data on patient demographics, comorbidities, type of surgery, clinical presentation, and postoperative complications were collected. DGE was defined according to the International Study Group of Pancreatic Surgery (ISGPS) criteria.

Results

Among the 97 patients, 50.5% were men, and the median age of the cohort was 65 years. Almost all patients underwent surgery for malignancy (96.9%). The classic Whipple group had fewer cases of DGE (9.8% vs. 30.6%, p=0.01), shorter hospital stays (7 vs. 11 days, p=0.001), and lower readmission rates (13.1% vs. 33.3%, p=0.017). The pylorus-sparing PD was associated with a lower incidence of bile leak [odds ratio (OR)=0.62, 95% confidence interval (CI): 0.53-0.73]. However, this technique was associated with a higher incidence of DGE (OR=4, 95% CI: 1.34-12.1), overall hospital admission rates (OR=3.3, 95% CI: 1.2-9.2), and admissions that resulted in a surgical event (OR=1.9, 95% CI: 1.21-2.96). DGE was associated with the need for a second surgery (OR=10.5, 95% CI: 2.8-39.5) and hospital readmission (OR=10, 95% CI: 3.1-32.3).

Conclusions

While the pylorus-sparing technique is associated with reduced bile leaks, it results in a higher incidence of DGE, prolonged hospital stays, and increased readmissions. Clinicians opting for the pylorus-sparing technique should ensure careful patient selection and rigorous postoperative monitoring.

## Introduction

Over the past few decades, hepato-pancreato-biliary surgery has undergone transformative innovations, leading to significant advancements in the safety, speed, precision, and overall effectiveness of these complex procedures [1]. This field witnessed significant innovation with the contributions of Allen Oldfather Whipple, who in 1935 performed the first “actual” pancreatoduodenectomy (PD), now known as Whipple’s procedure. His initial two-stage operation demonstrated a safer approach to pancreatic tumors, and he later refined it to a one-stage procedure including pancreatic resection, distal gastrectomy, and choledochoduodenostomy. Although he initially did not perform pancreatic anastomosis, this was included in subsequent procedures [2].

Whipple's technique revitalized surgical interest in treating pancreatic malignancies and provided hope to future generations. Early skepticism regarding Whipple’s procedure stemmed from anatomical and physiological misconceptions, such as the belief that the duodenum was essential for survival and that pancreatic juice flow was crucial. Initially, Whipple surgeries faced several challenges, including high rates of pancreatic fistula due to complications with pancreaticojejunal anastomosis; however, advancements in the field soon overcame these issues. In 1941, the Massachusetts General Hospital conducted the first "modern and standardized" PD, and over 2,000 Whipple procedures have been performed since then [3].

This surgical procedure has multiple variants, with classic PD and pylorus-sparing PD (Transverso-Longmire technique) being the two major types [4,5]. The former involves the resection of the head of the pancreas, the duodenum, the gallbladder, and part of the gastric tissue, while the latter preserves the pylorus, maintaining gastric continuity. In contemporary practice, pylorus-preserving PD (PPPD) has become the standard for treating tumors of the pancreatic head, duodenum, and distal cholangiocarcinomas. Postoperative complications of PD are common and may include pancreatic fistula, bile leaks, intra-abdominal collections, and bleeding [6-8].

Among these complications, delayed gastric emptying (DGE) stands out as one of the most significant due to its impact on patient recovery, prolonging hospital stay and increasing morbidity. DGE is defined as the inability of the stomach to empty its contents efficiently, which can result in symptoms such as nausea, vomiting, and abdominal distension. Various factors are associated with DGE and other adverse outcomes in patients undergoing PD [9-11]. These include the surgical technique employed, comorbidities such as diabetes mellitus and hypertension, as well as the need for additional interventions, such as total parenteral nutrition (TPN) and surgical reinterventions [11]. Additionally, the pylorus-sparing technique has been associated with a higher incidence of DGE than the classic technique, possibly due to the preservation of pyloric function and its influence on gastric motility [8]. Our study aims to analyze the association of the type of PD (classic Whipple vs. pylorus-sparing) with DGE and other adverse outcomes, by comprehensively comparing the two surgical techniques and their impact on postoperative recovery.

## Materials and methods

Study design

We conducted a retrospective and observational study involving adult patients of both genders who underwent classic PD (Whipple procedure) or pylorus-sparing PD (Transverso-Longmire technique) at Hospital Médica Sur between 2016 and 2021, performed by a single surgeon (CCh). Due to the study's retrospective nature, we obtained a waiver for informed consent. The study adhered to STROBE (Strengthening the Reporting of Observational Studies in Epidemiology) guidelines for reporting retrospective studies [12]. The study was approved by our institution's ethics and research committee. We strictly adhered to the tenets of the Declaration of Helsinki.

Selection criteria

We included men and women aged over 18 years who underwent either the Whipple procedure or pylorus-sparing technique (Transverso-Longmire) and had complete preoperative and postoperative follow-up (up to one year). We excluded patients who had early voluntary discharge, continued follow-ups at another institution, or had missing outcome information in their records.

Data extraction and variable definition

We collected patient demographic information (age, sex) and comorbidities (e.g., diabetes mellitus, hypertension, malignancy). We recorded the type of surgery the patient underwent (classic Whipple or pylorus-sparing PD) and the clinical presentation (e.g., jaundice). We gathered data on postoperative complications such as pancreatic fistula, bile leaks, intra-abdominal collection, and bleeding requiring transfusion support. Additionally, we collected data on ICU admission, hospital stay duration, mortality, use of TPN, readmissions, and surgical re-interventions, among other parameters at 90 days postoperatively. We defined DGE according to the International Study Group of Pancreatic Surgery (ISGPS) criteria, with confirmation of the absence of obstruction through imaging studies. Severe cases of DGE were reported.

Statistical analysis

We used SPSS Statistics v25 (IBM Corp., Armonk, NY) for statistical analysis. We employed the Kolmogorov-Smirnov test to assess the normality of numerical variables. We used classical descriptive statistics, such as median (range) and absolute frequency with percentages, to describe study variables. For comparative analysis, we separated the sample based on the type of surgery received (classic Whipple vs. pylorus-sparing PD). We compared study variables between groups using the Mann-Whitney U test or Pearson chi-square test, as appropriate. We calculated odds ratios (OR) with 95% confidence intervals (CI) and Spearman's bivariate correlations from two perspectives: first, by separating the sample based on the type of surgery received (e.g., analyzing whether the surgery was associated with DGE and other outcomes), and second, by separating the sample based on the presence or absence of DGE to understand which factors were associated with the presence of DGE. All tests were bivariate, and a p-value <0.05 was considered statistically significant. Missing values were not allowed in the analysis.

## Results

We included a total of 97 patients in the study. About half of these patients were men, and the average age was around 65 years (range: 28-85 years). The study found that nearly 28% of the patients had hypertension, and about 21% had diabetes mellitus. Most patients underwent PD due to malignancy. Additionally, those who had traditional PD were generally older than those who had the pylorus-sparing version (Table 1).

**Table 1:** Participant characteristics Characteristics of the study population and comparison by type of surgery (classic Whipple vs. pylorus-sparing pancreatoduodenectomy). Categorical variables were compared using the Chi-squared test. Age was compared using the Mann-Whitney U test. All variables except age are presented as frequency and percentage. Age is presented as average (range)

Variables	Total (n=97, 100%)		Classic Whipple (n=61, 62.88%)		Pylorus-sparing pancreatoduodenectomy (n=36, 37.12%)		P-value
Female	48	49.50%	30	49.20%	18	50.00%	
Male	49	50.50%	31	50.80%	18	50.00%	
Age	65	28–85	68	29–83	64	28–85	0.026
Systemic hypertension	27	27.80%	19	31.10%	8	22.20%	0.343
Diabetes mellitus	20	20.60%	16	26.20%	4	11.10%	0.075
Malignancy	94	96.90%	60	98.40%	34	94.40%	0.282
Jaundice	46	47.40%	24	39.30%	22	61.10%	0.038

The observed surgical complications included pancreatic fistula, bile leak, intra-abdominal collections, and bleeding. Almost half of the patients (41%) needed to be admitted to the ICU, where they stayed for an average of three days (range: 1-29 days). The average hospital stay was eight days (range: 3-90 days). DGE was seen in about 18% of patients. Reoperation and hospital readmission rates constituted approximately 12% and 21%, respectively. The primary reasons for readmission were severely delayed gastric emptying and abdominal collections, with most readmitted patients requiring additional surgical interventions (Table 2).

**Table 2:** Surgical outcomes Surgical complications, hospitalization duration, and other outcomes by type of surgery (classic Whipple vs. pylorus-sparing pancreatoduodenectomy). Severe bleeding was defined as the need for a blood transfusion. Categorical variables were compared using the Chi-squared test. Continuous variables were compared using the Mann-Whitney U test. All variables except ICU stay and hospitalization are presented as frequency and percentage. ICU stay and hospitalization are presented as average (range) ICU: intensive care unit

Variables	Total (n=97, 100%)		Classic Whipple (n=61, 62.88%)		Pylorus-sparing pancreatoduodenectomy (n=36, 37.12%)		P-value
Pancreatic fistula	42	43.30%	23	37.70%	19	52.80%	0.148
Bile leak	2	2.10%	2	3.30%	0	0.00%	0.272
Intra-abdominal collection	21	21.60%	10	16.40%	11	30.60%	0.102
Severe bleeding	7	7.20%	4	6.60%	3	8.30%	0.744
ICU admission	40	41.20%	23	37.70%	17	47.20%	0.358
ICU stay, days	3	1–29	2	1–29	3	1–22	0.742
Hospitalization, days	8	3–90	7	3–35	11	5–90	0.001
Death	2	2.10%	2	3.30%	0	0.00%	0.272
Delayed gastric emptying	17	17.50%	6	9.80%	11	30.60%	0.01
Total parenteral nutrition	18	18.60%	8	13.10%	10	27.80%	0.073
Reoperation	12	12.40%	5	8.20%	7	19.40%	0.104
Clavien-Dindo classification							
I	42	43.30%	31	50.80%	11	30.60%	0.145
II	4	4.10%	3	4.90%	1	2.80%	0.23
IIIA	10	10.30%	4	6.60%	6	16.70%	0.131
IIIB	1	1.00%	0	0.00%	1	2.80%	0.07
IV	40	41.20%	23	37.70%	17	47.20%	0.12
Readmission	20	20.60%	8	13.10%	12	33.30%	0.017
Surgical readmission	17	85.00%	8	100.00%	9	75.00%	0.125
Cause of readmission							
Severe delayed gastric emptying	7	35.00%	2	25.00%	5	41.70%	0.29
Surgical collections	7	35.00%	5	62.50%	2	16.70%	0.33
Anastomotic ulcers	1	5.00%	0	0.00%	1	8.30%	0.087
Infection	4	20.00%	1	12.50%	3	25.00%	0.43
Not associated with surgery	1	5.00%	0	0.00%	1	8.30%	0.101

**Figure 1:**
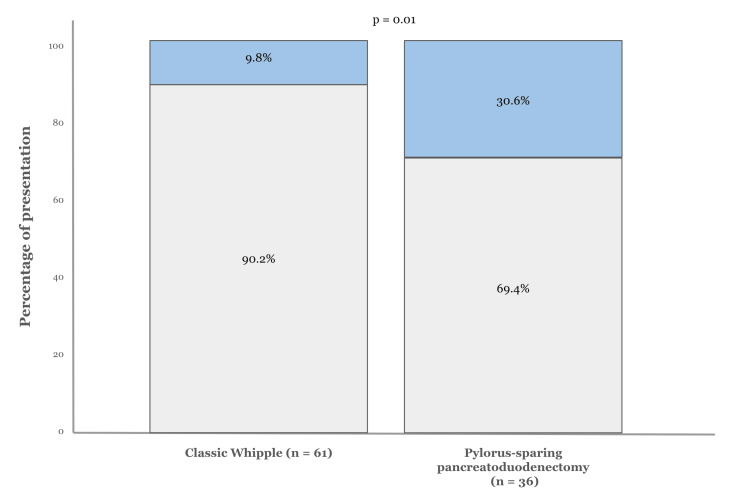
Proportion of patients with delayed gastric emptying by surgery type Bar graph demonstrating the percentage of delayed gastric emptying (blue box) in relation to the total number of patients who underwent each type of surgery (gray box). In classic Whipple, the studied complication was observed in 9.8%; in pylorus-sparing pancreatoduodenectomy, it was seen in 30.6% (p=0.01)

The rates of specific complications, including pancreatic fistula, bile leak, intra-abdominal collections, bleeding, ICU admission, and mortality, were similar between patients who had the classic Whipple procedure and those who underwent pylorus-sparing PD (Figure 1).

However, patients who had the classic Whipple procedure experienced fewer issues with DGE, fewer hospital readmissions, and shorter hospital stays compared to those who had the pylorus-sparing technique. Overall mortality was low, with only two deaths reported in the classic surgery group. Our analysis showed that while pylorus-sparing PD was associated with fewer bile leaks and deaths, it was linked to a higher incidence of DGE, overall hospital admissions, and admissions involving additional surgeries. Patients without comorbidities like hypertension and diabetes were less likely to experience DGE. On the other hand, DGE was strongly associated with the use of TPN, the need for a second surgery, and hospital readmission. Additionally, pylorus-sparing PD was notably linked to a higher incidence of DGE (Table 3).

**Table 3:** Associations and relationships studied Associations between the type of surgery, the presence of delayed gastric emptying, and other study variables CI: confidence interval; OR: odds ratio

	OR	95% CI
Pylorus-sparing pancreatoduodenectomy		
Pancreatic fistula	1.85	0.8–4.3
Bile leak	0.62	0.53–0.73
Intra-abdominal collection	2.2	0.84–6
Severe bleeding	1.3	0.3–6.1
ICU stay	1.5	0.64–3.4
Death	0.62	0.53–0.73
Delayed gastric emptying	4	1.34–12.1
Total parenteral nutrition	2.6	0.9–7.2
Reoperation	2.7	0.79–9.3
Readmission	3.3	1.2–9.2
Surgical readmission	1.9	1.21–2.96
Delayed gastric emptying		
Sex	0.85	0.3–2.4
Without systemic hypertension	0.8	0.66–0.87
Without diabetes mellitus	0.78	0.69–0.88
Surgery type	4	1.34–12.1
Total parenteral nutrition	88.7	17.8–439.9
Reoperation	10.5	2.8–39.5
Readmission	10	3.1–32.3
Surgical readmission	2.3	0.2–30

Additionally, pylorus-sparing PD moderately and positively correlated with the number of hospital days. The presence of DGE was positively associated with the number of hospital days and days in the ICU and showed a weak correlation with a higher grade in the Clavien-Dindo classification (Table 4).

**Table 4:** Correlations Correlations between surgery type, presence of delayed gastric emptying, and outcomes ICU: intensive care unit

	rho	P-value
Pylorus-sparing pancreatoduodenectomy		
ICU stay, days	0.053	0.747
Hospitalization, days	0.341	0.001
Clavien-Dindo classification	0.166	0.104
Delayed gastric emptying		
ICU stay, days	0.384	0.014
Hospitalization, days	0.506	0.0001
Clavien-Dindo classification	0.242	0.017

## Discussion

Our main finding is that although pylorus-sparing PD is associated with a lower incidence of bile leak and mortality compared to classic Whipple, it presents a higher rate of DGE, longer hospitalization, and higher readmission rates. Our findings highlight the importance of careful patient selection and rigorous postoperative monitoring for those undergoing the pylorus-sparing technique. Our findings show that 17.5% of patients who underwent PD experienced DGE, a significant complication impacting hospital length and readmission rates. This rate is considerably lower than the incidence of postoperative complications reported by Zubair et al. [9], who found that 50.5% of patients had complications like surgical site infections, anastomotic leaks, and pancreatic fistulas, with a mortality rate of 29.9%. However, our results align with the findings of Brown et al. [9], who reported a high incidence of long-term complications, including bile duct stenosis and pancreatitis, with 31.5% of patients having at least one long-term complication. Also, our data suggest that the pylorus-sparing technique, while associated with fewer bile leaks and mortality, presents a significantly higher risk of DGE, aligning with Brown et al.'s [8] findings linking pylorus preservation with long-term complications. 

In our study, DGE incidence was significantly higher in patients undergoing the pylorus-sparing technique than those who received classic Whipple. These results are consistent with Busquets et al. [11], who found a high incidence of DGE in the pylorus-sparing technique. However, they did not observe significant differences in postoperative morbidity and length of hospital stay between the two groups. Our study did identify significant differences in these outcomes, underscoring the importance of careful patient selection for the pylorus-sparing technique. Mirrielees et al. [13] highlight that DGE and pancreatic fistula are the most impactful complications following PD, which aligns with our findings; 17.5% of our patients experienced DGE, significantly impacting hospital length and readmission rates. Identifying these complications as priorities for quality improvement initiatives suggests that efforts to prevent DGE could significantly improve postoperative outcomes and reduce the burden on healthcare resources. Zdanowski et al. [14] and Sabogal et al. [15] identified several predictive factors for DGE after PD, including the surgical technique and preoperative factors such as opioid use and elevated bilirubin levels.

Our study also found that the pylorus-sparing technique was associated with a higher incidence of DGE, endorsing the conclusions of the above-mentioned studies. However, our results suggest that the absence of comorbidities such as hypertension and diabetes mellitus might have a protective effect against DGE, which was not assessed in the mentioned studies. Finally, Diener et al.'s [16] meta-analysis found no significant differences in mortality, morbidity, and survival between the pylorus-sparing and classic techniques. However, it highlighted a reduction in operating time and intraoperative blood loss with the pylorus-sparing technique. While our findings agree on the lack of significant differences in mortality, we identified a higher incidence of DGE and more extended hospital stay in the pylorus-sparing group, suggesting that despite intraoperative benefits, this technique may be associated with more frequent postoperative complications.

Our study has several limitations. For instance, the study's retrospective nature may have introduced biases in data collection and analysis, affecting the validity of our findings. Moreover, the smaller size of 97 means that the results may not be generalizable to a broader population. Also, all surgeries were performed by a single surgeon, which may limit the results' applicability to other clinical contexts with different levels of experience and surgical techniques. Finally, no multivariate analysis was conducted to adjust for potential confounding factors, which could influence the associations observed between the studied variables and clinical outcomes.

## Conclusions

This study highlights critical differences between classic Whipple surgery and pylorus-sparing PD. While both techniques exhibit similar overall complication and mortality rates, significant distinctions in postoperative outcomes were noted. Classic Whipple surgery resulted in fewer instances of DGE, shorter hospital stays, and reduced readmission rates compared to the pylorus-sparing approach. Conversely, pylorus-sparing surgery, though associated with lower bile leak and mortality rates, led to higher DGE incidence, extended hospitalizations, and more readmissions.

Our findings suggest that while pylorus-sparing PD may offer certain benefits, such as reduced bile leaks and mortality, it presents a higher risk of DGE, impacting recovery and resource utilization. The protective effect of comorbidities like hypertension and diabetes against DGE in our study further complicates the choice of surgical technique. It is important to emphasize that the decision between surgical techniques should be based on a thorough evaluation of patient-specific factors and expected postoperative outcomes. Further research with larger, multicenter cohorts is needed to refine these findings and optimize patient management strategies. Prioritizing efforts to prevent and manage DGE could improve recovery and reduce the burden on healthcare resources.
